# The Landscape of Connected Cancer Symptom Management in Rural America: A Narrative Review of Opportunities for Launching Connected Health Interventions

**DOI:** 10.13023/jah.0204.08

**Published:** 2020-11-17

**Authors:** Ming-Yuan Chih, Anna McCowan, Sadie Whittaker, Melinda Krakow, David K. Ahern, Eliah Aronoff-Spencer, Bradford W. Hesse, Timothy W. Mullett, Robin C. Vanderpool

**Affiliations:** University of Kentucky, mch266@uky.edu; University of Kentucky, almc264@g.uky.edu; ScienceOne, sadiew@science1.org; University of Mississippi Medical Center, mkrakow@umc.edu; Harvard Medical School, dahern@bwh.harvard.edu

**Keywords:** Appalachia, cancer, symptom management, broadband, Internet, telehealth, connected health, mobile health

## Abstract

**Background:**

The 2016 President’s Cancer Panel called for projects focusing on improving cancer symptom management using connected health technologies (broadband and telecommunications). However, rural communities, like those in Appalachia, may experience a “double burden” of high cancer rates and lower rates of broadband access and adoption necessary for connected health solutions.

**Purpose:**

To better understand the current landscape of connected health in the management of cancer symptoms in rural America.

**Methods:**

A literature search was conducted using four academic databases (PubMed, CINAHL, MEDLINE, and PsycINFO) to locate articles published from 2010 to 2019 relevant to connected cancer symptom management in rural America. Text screening was conducted to identify relevant publications.

**Results:**

Among 17 reviewed studies, four were conducted using a randomized controlled trial; the remainder were formative in design or small pilot projects. Five studies engaged stakeholders from rural communities in designing solutions. Most commonly studied symptoms were psychological/emotional symptoms, followed by physical symptoms, particularly pain. Technologies used were primarily telephone-based; few were Internet-enabled video conferencing or web-based. Advanced mobile and Internet-based approaches were generally in the development phase. Overall, both rural patients and healthcare providers reported high acceptance, usage, and satisfaction of connected health technologies. Ten of the 17 studies reported improved symptom management outcomes. Methodological challenges that limited the interpretation of the findings were summarized.

**Implications:**

The review identified a need to engage rural stakeholders to develop and test connected cancer symptom management solutions that are based on advanced mobile and broadband Internet technologies.

## INTRODUCTION

Cancer symptom management is an important area of research highlighted by the President’s Cancer Panel’s 2016 report on *“Improving Cancer-Related Outcomes with Connected Health”* and the Cancer Moonshot^SM^ Blue Ribbon Panel’s 2016 report.^1^,^2^ The National Cancer Institute (NCI) defines “symptom management” as “care given to improve the quality of life of patients who have a serious or life-threatening disease. The goal of symptom management is to prevent or treat, as early as possible, the symptoms of disease, side effects caused by treatment of a disease, and psychological, social, and spiritual problems related to a disease or its treatment.”^3^ Early and routine management of cancer symptoms and associated stressors can lead to improved treatment adherence, lower healthcare utilization, and reduced patient anxiety and depression.^4^ The Blue Ribbon Panel identified the need to accelerate development of evidence-based guidelines for “routine monitoring and management of patient-reported symptoms in all care settings and in all populations, throughout the cancer continuum.”^2^ To address this need, the President’s Cancer Panel proposed the use of connected health technologies to effectively manage cancer symptoms as part of routine cancer care.^1^

Connected health-enabled cancer symptom management refers to “use of broadband and telecommunications technologies to evaluate, diagnose, and monitor patients beyond the clinic”^5^ and encompasses a wide range of telecommunications approaches from traditional telephone-based support to advanced broadband Internet-enabled, web-based eHealth, and wireless Internet and mobile technologies.^5^,^6^ Connected health-enabled cancer symptom management can improve patient outcomes, including lower symptom burden, better quality of life, and longer survival.^7^–^9^ Connected health allows cancer patients to communicate their symptoms and receive care from their care teams without traveling to a traditional healthcare setting. Therefore, these approaches could especially benefit patients experiencing difficulty in accessing care because of their geographical location, such as those from the 13-state region of Appalachia.^10^,^11^

People living in rural communities, including Appalachia, experience health disparities, such as higher rates of cancer incidence and mortality, particularly in lung, prostate, and colorectal cancers.^12^,^13^ A similar trend was found related to the prevalence of cancer symptom burden,^14^ including physical, psychosocial, and financial distress.^15^,^16^ People living in rural areas also experience lower access to adequate broadband Internet, which enables connected health solutions.^17^,^18^ The realization that Appalachian communities have a “double burden” of high cancer rates and lower rates of broadband access and Internet adoption prompted the establishment of a public–private partnership called the L.A.U.N.C.H. (Linking and Amplifying User-Centered Network For Connected Health) Collaborative in 2017.^5^ This began a 3-year demonstration project focused on solving the issue of double burden faced by people living in rural Appalachian Kentucky.^5^

### Purpose

To inform the work in the L.A.U.N.C.H. Collaborative and future research in this area, an assessment of literature was conducted about the use of connected health technologies in symptom management among rural cancer patients in America over the past 10 years. A narrative review was then conducted to summarize a collection of original scientific studies from which narrative syntheses may be drawn to better understand the current field of research.^19^ Research questions that guided the selection of studies and evaluation of scientific content are: (1) What symptoms are the focus of connected health technologies developed for cancer symptom management in rural America?; (2) How and what connected health technologies for symptom management have been studied in this context over the last decade?; and (3) What evidence supports the feasibility and efficacy of using such an approach?

## METHODS

### Conceptual Model for Literature Search

To guide the literature search, the focus was on finding studies in the intersecting domains in the subject of interest: Internet/connected technology, rural populations, and symptom management in the context of cancer. A set of detailed search terms was developed for each conceptual domain (Appendix 1; see Additional Files). The terms “rural” and “Appalachia” were used to search literature focused on rural America. During the article screening, studies conducted in other countries were excluded to keep the focus on rural America. Symptom management search terms, such as “distress” and “side effects” focused the literature review on physical and psychosocial distress symptoms that patients experience as a result of their disease and treatments.^20^ Included were terms like “patient-reported outcomes” and “patient generated health data” to capture ways in which patients may report their symptoms and health-related data (e.g., heart rate) and could be useful for managing symptoms at home.^20^,^21^ Connected technologies such as “Internet” and “smartphone” were included in the search terms for Internet/connected technology. Specific terms about connected health technology, such as “telehealth,” “telemonitoring,” and “patient portal,” were used in the literature search as well.^22^ In this model, the literature falls under the interaction among all three domains in the context of cancer as the subject of interest in this review.

### Literature Review Process

The literature search was conducted in Spring 2020 to inventory current research on the topic of connected health technologies to support cancer symptom management in rural America over the last 10 years (2010–2019). General search terms were developed and used to derive specific subject headings in four academic literature databases: PubMed, CINAHL, MEDLINE, and APA PsycInfo (Appendix 1; see Additional Files). The general search terms and specific subject headings in each of the four domains were joined with the “OR” Boolean operator to capture all relevant articles in each domain. The search results in each domain were then joined with those from the other domains by using the “AND” Boolean operator to retrieve articles with relevancy in all domains.

**Figure 1:**
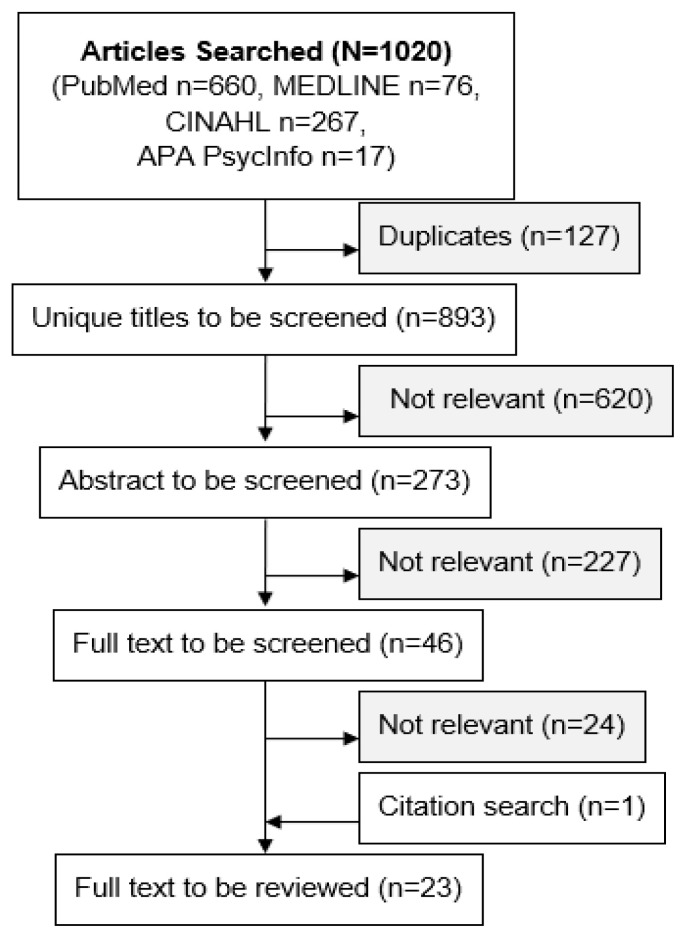
Flowchart of search and screening

Figure 1 shows the flowchart of the search and screening; 1020 articles were searched, and 23 full text articles were ultimately reviewed. The initial search was limited to English language publications between January 2010 and December 2019. Exclusion criteria included: abstracts, commentaries, reviews, international studies, and studies not focused on the rural cancer patient population, symptom management, or Internet/connected health technology. Three colleagues (MC, GP, AM) divided the screening tasks. At least two colleagues performed every screening task. Discrepancies were discussed and resolved to ensure the screening quality. The final articles to be reviewed were selected, and key information was retrieved through consensus. To answer the research questions outlined above, the following key information was retrieved:

1. Basic study information: the last name of the first author, journal title, publication year, study design, and rural cancer population
2. Cancer symptoms: psychosocial or physical symptoms and other needs/problems
3. Summary of rural cancer symptom management technology: type of Internet, information technology, symptom management program, and community ecosystem
4. Feasibility findings: acceptability, feasibility, usage, user satisfaction and challenges
5. Impact: patient outcomes, family/community outcomes, and healthcare/provider outcomes.

## RESULTS

The original screening yielded 22 selected articles. One paper^23^ reported the engagement outcomes of a study whose main outcomes were reported elsewhere,^24^ and the decision was to include it as well. Therefore, a total of 23 papers^23^–^45^ representing 17 unique studies were included in this review (Appendix 2; see Additional Files). Appendix 2 contains the summarized details of each of the 17 unique studies in these categories: study design, cancer population, symptoms, connected cancer symptom management system, feasibility, and key study impact. A synthesized summary of these results based on these 17 studies is provided below.

### Study Populations, Rural Settings, and Designs

All studies focused on evaluation of connected cancer symptom management among rural patients solely or as part of the overall study population. The studies represented a mix of tumor types and various rural areas across America. Two studies^30^,^37^ were conducted in Appalachia, with participants residing in West Virginia, Ohio, and Pennsylvania. Most connected symptom management programs were intended to be used in a patient’s home; however, Doorenbos (2010 and 2011)^27^,^28^ and Zhou (2016)^36^ developed telehealth and videoconferencing approaches that were partially deployed in rural clinics. Various study designs were employed, including five formative evaluation studies^28^,^31^,^38^,^39^,^42^; one cross-sectional survey^27^; four one-arm feasibility studies^36^,^40^,^41^,^43^; one two-arm, nonrandomized feasibility study^30^; two small pilot randomization controlled trials^29^,^37^; and four standard randomization controlled trials.^24^,^25^,^32^,^33^ Five studies^28^,^31^,^38^,^39^,^42^ used participatory design approaches to solicit input from stakeholders to involve them in the design of connected health solutions that were more culturally informed.

### Cancer Symptoms, Side Effects, and Needs Managed by Connected Health in Rural America

The most common cancer symptoms targeted by connected health interventions were psychological/emotional symptoms, fatigue, loss of physical function/restricted abilities, and pain (Table 1). These are not dissimilar to symptoms experienced by patients living outside of rural communities; however, limited access to care suggests that the symptom burden experienced by rural patients may be unique. Other symptoms that are the focus of current connected health solutions developed for symptom management include dyspnea and coughing, loss of appetite, nausea, and vomiting (Table 1). Along with these disease specific symptoms, management interventions also focused on financial and spiritual needs and medication adherence.

### Connected Cancer Symptom Management in Rural America

Most reviewed symptom management approaches offered both remote symptom assessment and symptom management capabilities. Researchers reported using different data sources to assess symptoms that were separate from how other health data were captured. The primary source reported for remote cancer symptom data was patients’ self-report collected via communications technologies, such as interactive voice-response systems,^25^ telemonitors,^30^,^37^ videoconferences,^27^,^28^,^40^ e-mails,^33^ web-based systems,^39^,^41^,^42^ and smartphone apps.^38^ Another data source was direct clinician assessment via providers’ telephone calls to patients^24^,^29^,^32^,^33^,^43^ and video conferencing.^27^,^28^,^40^ The telemonitoring systems tested in Petitte (2014) and Chen (2016) also collected objective health data from peripheral sensors (e.g., blood pressure monitor).^30^,^37^ Researchers used the collected symptom data to guide the symptom management programs delivered to patients. Either clinician-delivered or web-based systems provided these symptom management programs. Thirteen clinician-delivered remote symptom management programs were conducted at a set schedule via telephone calls^24^,^25^,^29^,^30^,^32^,^37^ or video conferencing.^27^,^28^,^33^,^36^,^40^,^42^,^43^ Six web-based symptom management systems were made available at any time via Internet-enabled computers and mobile devices.^24^,^31^,^38^,^39^,^41^,^42^ All six web-based symptom management systems offered patient education information on cancer symptoms, coping techniques, or self-management skills. In addition, one^24^ of them provided an online forum for social support. The clinician-delivered programs provided not only tailored patient education (similar to web-based systems but at a set schedule and not available at any time), but also care services, such as care management and problem solving,^25^,^29^,^40^ that can only be done through interaction with clinicians. Overall, more recent intervention programs adopted advanced information and communications technologies, such as mobile apps, to deliver symptom management support to rural cancer patients over the Internet. However, among the studies^24^,^31^,^38^ that mentioned the development of advanced mobile apps to be used on tablets and smartphones, only one^24^ developed and tested an actual system. Only one study mentioned the use of pedometers to track steps,^33^–^35^ but the pedometer used in this study was unlikely to be a wireless connected wearable (e.g., Fitbit), because researchers asked the participants to report their steps manually.

### Testing Feasibility of Symptom Management Approaches

Most researchers sought to understand the feasibility of operating a technology-focused intervention for symptom management with a rural cancer population. All but one study^31^ conducted or reported some form of feasibility of the respective cancer symptom management systems. The feasibility measures reported in these studies included recruitment,^24^,^29^,^30^,^33^,^40^ retention,^25^,^29^ satisfaction,^27^,^29^,^36^,^37^,^38^ ease of use,^42^ usefulness,^27^,^38^,^42^ willingness to use,^30^,^39^ technology availability and acceptance,^40^,^42^ study completion,^29^,^30^,^34^,^36^,^39^–^41^,^43^ system usage,^24^,^29^,^32^,^33^,^37^,^39^,^41^,^42^,^43^ and costs.^26^,^33^,^37^ Most studies either required the participants to have their own access to the Internet and/or needed devices,^24^,^25^,^29^,^32^,^34^,^38^–^42^ or provided the participants with access to these technologies directly^30^,^36^,^37^ or via community clinics.^27^,^28^ However, two studies reported that having no Internet access caused problems in recruitment.^24^,^30^ Three studies^36^,^37^,^42^ reported the access to and quality of the Internet connectivity in rural areas is often challenging based on the participants’ feedback.

### Impact and Key Findings

Ten studies reported improved patient health outcomes (e.g., improved symptoms, functional status, healthy behaviors and quality of life) among those with access to a cancer symptom management system.^24^,^25^,^29^,^30^,^33^,^36^,^37^,^40^,^41^,^43^ However, the interpretation of these findings needs to consider the variations in study design (e.g., feasibility^30^,^36^,^40^,^41^,^43^,^29^,^37^ vs. efficacy focused^24^,^25^,^33^). Researchers in one study did not find the significant improvement in stress reduction among those receiving their online video conferencing group education program.^36^ They attributed this finding to the insufficient intervention doses (i.e., four shortened online sessions as compared to 10 in-person therapy sessions) and the challenges of using this novel technology (e.g., distraction during a videoconference and hardware/software/unstable connection issues).^36^ Three studies reported that the connected symptom management may likely improve healthcare delivery, including reduced utilization of physical therapy services,^29^ increased access to care,^28^ and increased completion rates and adherence to planned cancer therapies.^41^ Only two studies^26^,^33^ reported incremental cost-effectiveness ratios and concluded that their connected cancer symptom management systems were cost effective.

## IMPLICATIONS

A narrative review of 17 studies (23 papers) was synthesized; this focused on connected cancer symptom management in rural America. Several key implications can be derived from the results to inform future research. Based on the reviewed studies, cancer patients and survivors in rural America have a positive assessment of how connected health can improve access to care and self-management. These studies assessed some element of patient, survivor, or caregiver receptivity and usage of connected health. In these assessments, a majority of stakeholders showed positive receptivity to connected health, meaning these studies suggest rural cancer patients, survivors, and caregivers are open to use of technology as an element of their care when it enables remote support. Overall, successful recruitment and study completion indicate that connected health-enabled cancer symptom management in rural settings are achievable. The improved patient and healthcare delivery outcomes warrant further research. However, current evidence regarding the impact of connected cancer symptom management is weak due to the fact that most reviewed studies in this area are early phase feasibility evaluations. The larger randomized controlled trials often included nonrural patients and did not separate analyses results by rural status. There is a real need for rigorous experimental studies in this field.

In the last decade, mobile and broadband Internet have become part of many Americans’ daily life.^46^,^47^ However, people living in rural areas with insufficient access to primary care may also not have adequate access to the broadband Internet that enables telehealth visits.^17^ Some of the reviewed studies reported similar concerns of inadequate access to the Internet. Moreover, in this review, most studies focused on traditional telehealth approaches using telephone-based connectivity. A few studies aimed at using advanced mobile and broadband Internet technology were mostly in the development phase. The Society of Behavioral Medicine has recently urged nationwide efforts to expand the “access to high-speed, high-definition internet and increasing broadband width for rural communities in the USA to increase telehealth opportunities for populations facing geographic barriers to accessing quality healthcare.”^18 (p489)^ Projects aimed to develop and test connected symptom management approaches based on advanced mobile and broadband Internet technology will offer the lessons learned and evidence needed to strengthen our efforts as a nation to improve the access and adoption of broadband Internet and provide connected health for rural America.

The symptom burden of cancer patients living in rural communities, and the requirements for connected health systems to manage symptoms in these settings, differ from urban populations. One example is the logistical challenge of living far away from the cancer center as described by Zhou (2016).^36^ Lack of access to healthcare providers in rural areas can lead to difficulty in getting adequate care. Such restrictions to access can have a profound impact on symptom burden for patients living in rural settings, which can adversely affect medical outcomes. The interventions reviewed in these studies were aimed at remotely alleviating symptoms and side effects that rural patients experience, in an attempt to lessen the double burden that rural patients carry.

As with any setting, there were specific cultural and communication differences evident in rural settings that presented unique challenges and opportunities in the research of connected cancer symptom management systems.^28^,^30^ Also, we recognize that the cost of broadband services and technologies is a barrier to adoption of connected cancer symptom management systems. One potential resource is the Federal Communications Commission’s (FCC) Lifeline program, which provides low income consumers with access to broadband at a low cost.^48^ Partnering with those who will eventually use an intervention can ensure its success and longevity. This review indicates that the research team and the symptom management approaches it is developing need to be trusted and fit in the unique social environment, especially in the rural areas.^39^ To achieve this, future researchers need to understand the pace of life, priorities, assets, communication styles, and local conventions to truly partner with people in rural communities.^28^,^49^

Tarver and Haggstrom (2019) recently published a systematic review on the use of cancer-specific, emerging Internet technologies among underserved populations.^22^ Their review included 71 articles, among which 14 focused on rural populations and published in earlier years (1995–2016). Moreover, they included the systems designed for cancer screening (e.g., telegenetics counseling), which was not the focus of this review. Likely due to these differences in the scope and inclusion criteria, we were able to locate and review a different set of articles with only two articles overlapping with theirs.^25^,^27^ This review retrieved more detailed information about symptom management approaches and described the impact and key findings from the feasibility of the approaches. Despite these differences, both reviews have found that connected health technologies are generally feasible and acceptable among rural and underserved populations.

Two strengths of this review are worth mentioning. First, relevant studies of the last 10 years that focused on a very specific topic, namely cancer symptom management in rural America using connected health technologies, were searched and summarized. Useful information was retrieved, including most commonly experienced cancer symptoms, which connected symptom management approaches have been tested, and their related feasibility and impact. This provides an overview of the current landscape and identifies gaps to inform future research.

There are also limitations. This is not a systematic review; it is a narrative review that can be viewed as formative research. The results from this narrative review may not be comprehensive and generalizable. There was no attempt to evaluate or rate the methodological quality of each study; consequently, the results may be limited by the variation in experimental control and rigor used across the studies reviewed. Second, because of the search keywords and coding categories, it is likely that relevant articles or information in the review articles may have been missed. We have discussed the results among authors and updated the search and coding methods in several iterations to ensure the completeness of the review.

Several known challenges include the aforementioned cultural sensitivity, the scarcity of research testing advanced Internet and mobile technologies, and the initial investment costs.^26^,^33^,^37^ These challenges call for innovative solutions to support symptom management among cancer patients living in rural settings. Appalachia is an example of such a setting where many patients may live further from clinics. Connected health solutions that necessitate access to advanced Internet or broadband and mobile technologies may have the potential to significantly improve symptom management in cancer patients, resulting in improved outcomes. As new opportunities arise for telehealth reimbursements,^50^ future research is needed on how connected cancer symptom management can become an integral part of rural cancer care. In its demonstration project, the L.A.U.N.C.H. Collaborative is adopting a community-based approach^48^,^51^ to co-design broadband Internet-enabled cancer symptom management solutions with the Appalachian community that we hope will improve the lives of those experiencing cancer.

**SUMMARY BOX**

**What is already known on this topic?** Early and routine management of cancer symptoms and associated stressors can lead to improved treatment adherence, lower healthcare utilization, and reduced patient distress. Appalachian communities may experience a “double burden” of high cancer rates and lower rates of broadband access and adoption necessary for connected health-enabled cancer symptom management.

**What is added by this report?** Rural cancer patients are receptive and accepting towards connected health technology, which could bridge the gap between symptom management and associated challenges in rural areas. However, few studies showed efficacy outcomes, and few tested advanced Internet and mobile communication technologies.

**What are the implications for future research?** This review highlights the need for more rigorous studies involving rural communities in the development and testing of broadband-enabled connected systems to support cancer symptom management.

## Figures and Tables

**Table 1:** Cancer Symptoms/Needs Reported by Rural Patients and Managed via Connected Health

Symptoms/Needs	Studies reported in	Cancer type studied
Psychological/emotional: depression and/or anxiety	10 [24,25,27,30–32,36,37,39,40]	Mixed
Pain	7 [25,27,28, 30,32,37,42]	Mixed
Loss of normal physical function	5 [24,29,30,37,41]	Mixed
Dyspnea, coughing	2 [30,37]	Lung cancer
Fatigue	7 [24,29,30,32,37, 40,42]	Mixed
Loss of appetite	4 [30,37,41,42]	Mixed
Nausea and vomiting	3 [30,37,41]	Mixed
Insomnia	2 [40,42]	Mixed
Lymphedema	1 [32]	Breast cancer
Difficulty standing and/or walking	2 [30,37]	Lung cancer
Weight gain	2 [34,43]	Breast cancer
Financial/spiritual needs	2 [31,32]	Breast cancer
Medication adherence	1 [25]	Mixed
Vital signs	2 [30,37]	Lung cancer

## APPENDIX 1 SEARCH TERMS

### A. General search terms

- Rural Population: Appalachian OR Appalachia OR rural
- Symptoms: symptom OR emotion OR signs OR distress OR “patient reported outcome” OR “patient generated health data” OR “side effects”
- Connected Technology: “information technology” OR computer OR laptop OR desktop OR smartphone OR “smart phone” OR “cell phone” OR “cellular phone” OR Internet OR web OR website OR ehealth OR “e-health” OR mhealth OR “m-health” OR telehealth OR telemedicine OR telemonitoring OR telecommunication OR videoconferencing OR “text message” OR SMS OR “patient portal”
- Cancer: Cancer or Neoplasms

### B. Database-specific subject headings

#### PubMed

- Rural Population: “Appalachian region”[Mesh] OR “rural population”[Mesh] OR “rural health services”[Mesh] OR “rural health”[Mesh] OR “hospitals, rural”[Mesh]
- Symptom Management: “patient outcome assessment”[Mesh] OR “vital signs”[Mesh] OR “symptom assessment”[Mesh] OR “behavioral symptoms”[Mesh] OR “emotions”[Mesh] OR “pathological conditions, signs and symptoms”[Mesh] OR “Drug-Related Side Effects and Adverse Reactions”[Mesh] OR “Radiation Effects”[Mesh] OR “Patient Reported Outcome Measures”[Mesh] OR “Patient Generated Health Data”[Mesh]
- Connected Technology: “computer systems”[Mesh] OR “software”[Mesh] OR “communications media”[Mesh] OR “information technology”[Mesh] OR “telecommunications”[Mesh] OR “internet”[Mesh] OR “Patient Portals”[Mesh]

#### CINAHL

- Rural Population: (MH “Appalachian Region+”) OR MH “Rural Health Centers”) OR (MH “Hospitals, Rural”) OR (MH “Rural Population”) OR (MH “Rural Health Services”) OR (MH “Rural Health Nursing”) OR (MH “Rural Areas”) OR (MH “Rural Health”) OR (MH “Frontier Nursing Service”)
- Symptom Management: (MH “Behavioral Symptoms+”) OR (MH “Symptoms and General Pathology+”) OR (MH “Symptoms+”) OR (MH “Emotions+”) OR (MH “Stress, Psychological+”) OR (MH “Patient-Reported Outcomes”) OR (MH “Outcome Assessment”) OR (MH “Symptom Distress”) OR (MH “Vital Signs+”) OR (MH “Medication Side Effects (Saba CCC)”)
- Connected Technology: (MH “Computer Communication Networks+”) OR (MH “Computer Systems+”) OR (MH “Telecommunications+”) OR (MH “Information Technology+”)

#### MEDLINE

- Rural Population: (MH “Rural Health Services+”) OR (MH “Rural Population”) OR (MH “Rural Nursing”)OR (MH “Hospitals, Rural”) OR (MH “Rural Health”) OR (MH “Appalachian Region+”)
- Symptom Management: (MH “Behavioral Symptoms+”) OR (MH “Symptom Assessment”) OR (MH “Signs and Symptoms+”) OR (MH ““Vital Signs+”) OR (MH “Emotions+”) OR (MH “Radiation Effects+”) OR (MH “Drug-Related Side Effects and Adverse Reactions+”) OR (MH “Patient-Reported Outcome Measures+”) OR (MH “Patient Outcome Assessment”)
- Connected Technology: (MH “Computer Communication Networks+”) OR (MH “Computer Systems+”) OR (MH “Telecommunications+”) OR (MH “Information Technology+”)

#### APA PsycInfo

- Rural Population: DE “Rural Environments”
- Symptom Management: DE “Symptoms” OR DE “Major Depression” OR DE “Distress” OR DE “Emotional States” OR DE “Patient Reported Outcome Measures” OR DE “Side Effects (Treatment)” OR DE “Side Effects (Drug)”
- Connected Technology: DE “Digital Interventions” OR DE “Computer Mediated Communication” OR DE “Speech Processing (Mechanical)” OR DE “Websites” OR DE “Blog” OR DE “Telepsychology” OR DE “Telepsychiatry” OR DE “Telemedicine” OR DE “Teleconferencing” OR DE “Online Therapy” OR DE “Teleconsultation” OR DE “Telerehabilitation” OR DE “Assistive Technology” OR DE “Medical Therapeutic Devices” OR DE “Information Systems” OR DE “Internet” OR DE “Information and Communication Technology” OR DE “Automated Information Processing” OR DE “Digital Technology” OR DE “Health Information Technology” OR DE “Wireless Technologies” OR DE “Health Information Technology” OR DE “Telemetry” OR DE “Electronic Health Services” OR DE “Digital Interventions” OR DE “Mobile Health” OR DE “Precision Medicine” OR DE “Telemedicine” OR DE “Wearable Devices” OR DE “Wireless Technologies” OR DE “Mobile Technology” OR DE “Text Messaging”

## Appendix 2 Summary of Connected Cancer Symptom Management Literature

**Table:**

Author (Year), Journal	Study Design	Rural Cancer Population	Symptoms, side effects, and needs	Summary of Connected Rural Cancer Symptom Management System	Feasibility	Impact and Key Findings
1. Kroenke (2010), JAMA^25^ Yoo (2014), General Hospital Psychiatry^26^	12-month, 2-arm Indiana Cancer Pain and Depression (INCPAD) Randomized Controlled Trial (RCT)	Rural cancer patients (n=405) with depression, pain, or both, recruited from 16 cancer provider organizations, including 10 serving rural Indiana. Mixed cancer types include breast, lung, gastrointestinal, hematologic, genitourinary and others.	Pain, depression, adverse effects, medication adherence, global improvement	Weekly automated home-based symptom monitoring via either interactive voice calls or web-based surveys with frequency from twice a week for the first 3 weeks to once a month in the last 6 months was provided. Telephonic care management was delivered by a nurse-physician team: 3 scheduled calls (1, 4, and 12 weeks) and triggered calls by responses from the symptom monitoring mentioned above. No Internet implication was mentioned. Participants were not offered Internet or devices.	The recruitment rate was 65.7% (405/616). During the study period, patients received on average 11.2 care manager calls and 20.5 automated system monitoring contacts. Care managers spent 157 minutes per patient on the calls. The retention rate at 12 months is 66%. It is feasible to provide telephone-based, technology-augmented symptom management to both urban and rural populations.^25^ The total costs of INCPAD is $1189 per patient at the start up. After which, the total costs per patient decreased to $818.^26^	Improved pain (ES=0.36–0.67) and depression severity (ES=0.31–0.45) and other quality of life domains (e.g., mental health, physical symptom burden) were found over the study period.^25^ Varied Incremental Cost-Effectiveness Ratios (ICERs) were reported based on different outcome measures. Most ICERs were under or comparable with that of other disease management interventions.^26^
2. Doorenbos (2010), Clinical Journal of Oncology Nursing^27^	Cross-sectional, descriptive study	32 American Indians (AI) and Alaskan Native (AN) breast (34%), lung (28%) and other cancer survivors. 27 had a diagnosis of late-stage cancers	Pain and psychological symptoms, end-of-life issues, and other needs	A total of 12, 2-hour monthly virtual support group meetings were conducted using Internet-enabled telehealth systems hosted at 25 remote rural tribal sites in Washington and Alaska. Participants, including survivors, families, and providers) connected virtually discussed cancer related learning materials. There is no mention of participant’s own access to the Internet.	The recruitment rate is 64% (32/50). A high level of satisfaction was reported by cancer survivors. The top three highest reported satisfaction points were the opportunity to interact with survivors from other AI and AN tribal communities, useful information, and telehealth.	No impact on health was reported. Other key findings contain a list of identified information needs including nutrition, side effects during treatment, cancer education, and culture-related issues.
3. Doorenbos (2011), Tele-medicine and e-Health 28	Participatory formative evaluation to develop a regional telehealth network	Clinical sites serving AI and AN in Washington, Montana, and Alaska	Pain and psychological symptoms, end of life issues, and other needs	The Native People for Cancer Control Telehealth Network (NPCCTN) provided cancer-related educational sessions, consultative services to providers and monthly support groups, education activities, and telecare for cancer patients, families, and their healthcare providers. There is no mention of participant’s own access to the Internet.	Between 2006 and 2009, 9 tribal clinics in Washington and 26 clinical sites in Alaska participated in the NPCCTN.	Twenty-seven cancer education presentations were attended by 369 providers. 44 case conferences were held to discuss 129 cases. 513 patient encounters (e.g., tele-dermatology consults) were conducted.
4. Hegel (2011) Psycho-Oncology 29	A pilot RCT, the Living Well trial, to study the feasibility of a telephonic problem-solving/occupational therapy program	Rural breast cancer patients (n=31) receiving chemotherapy and living in Lebanon, New Hampshire were recruited from the breast oncology clinic at Dartmouth-Hitchcock Medical Center	Participation restriction, fatigue, stress, nutrition, sleep problems	Six weekly sessions of Telephone delivered Problem Solving and Occupational Therapy (PST-OT) intervention to improve participation restrictions (i.e., the limitations interfering with daily lives) in rural breast cancer patients undergoing chemotherapy. It is assumed everyone has access to a telephone. There is no mention of the Internet.	The recruitment rate was 67% (37/46). The retention rate was 81% with 6 dropouts. 24 (77%) completed all assessments. Over 90% were highly satisfied with PST-OT and considered it to be helpful for overcoming participation restrictions. 97% of PST-OT sessions were completed. An average of 2.6 problems were addressed over the 6-session treatment.	Small improvements in social, emotional, and functional wellbeing were noted (ES=0.086–0.112) at the 6-week follow up but not at the 12-week follow up. A trending (p<0.06) reduction of physical therapy service utilization was also noted.
5. Befort (2012) Breast Cancer Research and Treatment^43^	One-arm treatment study assessing the impact of a weight loss intervention delivered via conference call technology	Rural breast cancer survivors (n=35) recruited from three rural cancer centers in Kansas	Being overweight/obese. The intervention targeted weight loss and maintaining the weight lost	Six-month group phone-based weight loss intervention involved weekly 60-min conference call sessions. Topics discussed included progress towards goals and a diet, physical activity, survivorship, or behavioral topic of the week. Unlike the later study by 9. Befort (2016), it did not involve a Phase 2 or newsletter comparison. Eligible participants were contacted by phone, so it is assumed everyone has access to a telephone. There is no mention of the Internet.	The recruitment rate was 83% (35/42). 91% of participants completed the study and attended >75% of intervention phone conferences. 71% of participants who completed the program met the goal of 225 minutes of physical exercise per week. Conference call interventions provide the support and benefits of group efforts to remote and/or isolated locations.	As compared to baseline, statistically significant changes were found in weight loss, physical activity, diet, depression, and body image subscales. Participants were found to feel stronger, healthier, and like they had more subjective control over their health following the intervention.
6. Petitte (2014) Oncol Nurs Forum 30	Exploratory, descriptive, and observational, two-group comparison feasibility study	Rural patients from West Virginia (n=10) hospitalized with **lung cancer** as primary or secondary-related diagnosis. Recruited from hospital at discharge.	Subjective measures: Pain, nausea/vomiting, dyspnea, fatigue, limited activity, coughing, standing, and walking, anxiety and upset, and appetite; Objective measures: Temperature, pulse rate, blood pressure, weight, oxygen saturation	Use of Genesis DM Class II telemonitoring device from Honeywell HomMed set up at home for 14 days with daily phone calls from nurse coaching. The device transmitted data electronically via patient’s landlines or a wireless General Packet Radio Service (GPRS). Researchers were able to lease a telemonitor with a modem attached for data transmission; one participant was unable to complete the study because they did not have a telephone available at their rural residence.	Of 20 eligible patients, 10 (50%) consented. Among them, 1 of 5 (20%) usual care and 3 of 5 (60%) monitored patients completed the study. Telemonitored data transmission was feasible in rural areas with high satisfaction. Telemonitoring was able to collect physiological data. Patients were willing to utilize the telephone to report symptoms to researchers.	Nurses were able to use the information collected as useful indicators of patient risk and for motivational interviewing and assisted the nurse in their knowledge to coach patents. Challenges include environment, culture, technology, and overall enrollment/retention.
7. Dahlke (2015), Journal of Cancer Education^31^	A project to develop a mobile and web-based resource tool to support cancer survivors	10 site visits of community-based oncology practices at both urban and rural settings, a retreat with 150 cancer survivors and providers, and follow up polling and webinars.	Financial, spiritual, emotional, nutritional, and physical needs, community-specific needs of cancer survivors	NaviCanPlan provides a collection of nine information resource categories, including personal services, physical activity, community support, financial, legal or insurance, counseling, nutritional, spiritual/faith-based, general medical, and cancer centers. NaviCanPlan shows these resources on a map interface for easy navigation.	There was no data reported about the feasibility of this system.	This study showed the needs for a programmatic approach to education and resources in the communities to support cancer survivors.
8. Pisu (2015), Journal of Cancer Survivorship: Research and Practice 32	A 2-arm RCT comparing the early vs. delayed education approach of the Rural Breast Cancer Survivors (RBCS) intervention	Breast cancer survivors (n=328) in rural Florida.	ED #1 (First education call): Fatigue, lymphedema, pain, menopausal symptoms ED #2: Healthy lifestyle behaviors, relationships, work/financial challenges ED #3: Anxiety, depression, fear, spiritual changes	The 12-month telephone delivered RBCS intervention includes four components: Initial assessment, three education calls, a follow-up education call, and six support calls. Written education materials include a 140-page educational manual and 38 activity sheets. Having access to telephone or cellphone is an eligibility criterion.	On average, calls lasted between 25 and 67 minutes with the intake assessment calls as the longest and the follow-up education as the shortest. Documentation took about 18.4 minutes on average.	Patients with higher depressive symptoms, who used other support, and who received education calls first (the early education group), spent more time with the interventionists in the initial assessment and education calls.
9. Befort (2016), Obesity (Silver Spring, Md.)^33^ Befort (2014), Contemporary Clinical Trials 34 Christifano (2016), Nutrition & Cancer 44 Fazzino (2016), Supportive Care in Cancer 45 Fazzino (2017), Obesity 35	Protocol reporting a RCT designed to examine two alternatives for delivering extended care for weight loss maintenance (group phone counseling vs. newsletter comparison)	Female breast cancer survivors (n=216) residing in a rural area	Being overweight/obese, intervention targeted weight loss and maintaining the weight lost	The six-month group phone-based weight loss intervention (Phase 1) involved weekly 60-min conference call sessions. Phase 2 included women who lost at least 5% of entry weight and focused on maintenance through continued group phone counseling for 12 months (26 weeks). A self-monitoring report (weight, daily food intake, physical activity minutes/steps) was sent by the participants to their group leader weekly by voice message, email, or fax. Befort (2016) includes a quote suggesting rural households have low broadband access. It is assumed everyone had access to a telephone. There is no mention of the Internet needed for intervention.	721 women were screened by phone. Among them, 216 (30%) met the eligibility and attended a study-oriented visit, of which 210 (97%) completed baseline testing and enrolled. Among 210 in Phase 1, 172 (82%) participants lost greater than or equal to 5% of their starting weight and were subsequently enrolled in the RCT (Phase 2). Group phone counseling included 60% of participants attending >75% of sessions. The study retention rates were 87% for the intervention group and 92% in the control group.^33^–^34^ Accountability, group support, diet convenience, effort levels, and motivation to physical activity were identified as the key to success. Most showed interest in paying to continue the program.^45^	Weight loss was significantly greater (p=0.03) and more likely to maintain (p=0.02) in group phone counseling as compared to a newsletter approach).^33^ Participants’ diet quality (ES=0.4–1) and weight loss (ES=2.2) was also improved during the first 6-month group phone phase.^44^ Significantly increased physical activity during the 6-month group phone-based intervention was reported and maintained during maintenance phase.^35^ From the providers’ perspective, ICERs (between groups) were $118 to avoid 1kg of weight gain, $1,116 to one person within 3% of her 6-month weight and $882 to keep one person 5% or more below her baseline weight. ICERS increased to $422, $3992, and $3155 after adding participants’ costs, i.e., their time spent in the program activities.^33^
10. Zhou (2016), Rural and Remote Health 36	A pilot study testing the feasibility of a remote access video conferencing educational program aimed at group cognitive-behavioral stress management for patients.	Breast, prostate, and blood cancer survivors (n=16), aged 24–70, were from Massachusetts, Rhode Island, Maine, and New Hampshire. All but one participant said their commute to the cancer center was 30+ minutes.	Stress/mental health	The videoconference group education program included 4 one-hour weekly sessions about cognitive-behavioral stress management (CBSM). The first meeting for participants was in-person in the clinic, and they received a tablet (iPad) to attend subsequent group video conferences via Cisco WebEx. Program-related homework and practice exercises were provided via eBook for participants to complete between sessions. A trial connection was done a day before each session to resolve network and technical issues. Participants provided with a loaner iPad. There is no mention of participant Internet status.	87.5% (14/16) actually completed all sessions. Main reasons for joining the program included: not having to attend in person (n=10), preferred video conference (n=5) and avoid traffic (n=3). Benefits included helpful program (n=10), suitable contents for improving stress (n=8), effective facilitation (n=5). Qualitative feedback includes being helpful (n=10), good contents (n=8), and effective facilitation (n=5) Challenges included developing a bond with other survivors and experiencing distraction and technical difficulty (connection). Some patients (n=4) thought the program was too brief.	A pre- and post-test comparison showed modest but not statistically significant improvement on psychological stress. Group-based communication between sessions can also be used to encourage connections between program participants.
11. Chen (2016) International Journal of Chronic Diseases and Therapy 37	A 2-arm pilot RCT comparing Tele-monitoring vs. usual care with a 14-day intervention period and 60-day post discharge follow up.	The majority (>90%) of study patients (n=47) resided in rural Appalachian communities in West Virginia, Ohio, and Pennsylvania	Subjective measures: Pain, nausea/vomiting, dyspnea, fatigue, limited activity, coughing, difficulty in standing and walking, anxiety and feeling upset, and decreased appetite; Objective measures: Temperature, pulse rate, blood pressure, weight, pulse oximeter oxygen saturation	The fourteen-day wireless, in-home telemonitoring system (Honeywell HomMed Genesis DM) and patient-centered phone coaching by nurses aimed at developing self-management skills Telemonitor devices were set either in conjunction with the home landline or if unavailable supplied with a wireless communicator.	The recruitment rate was 42%. The study completion rate was 51% (24/47). About 60% were compliant with data reporting. Telemonitoring group patients were more satisfied with the telemonitoring system, the care they received and more compliant with the study protocol than control group participants. Although conveying wireless data in mountainous remote surroundings was challenging, the feasibility of technology use and the at-home intervention was confirmed and widely accepted.	Both functional status (Wald X^2^=3.78, p=0.05) and QOL (Wald X^2^=7.25, p=0.003) of the intervention group had consistent improvement over time. The telemonitoring group reported more calls. outpatient visits or ER visits but fewer rehospitalization. When considering compliance, telemonitoring compliance participants has the least number of ER visits and rehospitalization. However, healthcare utilization and costs did not differ between groups likely due to a small sample size. No cost-effectiveness ratio was reported.
12. Baseman (2017) JMIR Cancer 38	A prototyping pilot study to explore the feasibility and acceptability of a mobile health survivorship care app	Six “urban” Breast cancer survivors and five providers (4 primary care and 1 oncology) who have experiences treating rural patients	Survivor reported symptoms	A clickable prototype of the SmartSurvivor mobile app was developed based on the Institute of Medicine (IOM)-Recommended Survivorship Care Plan (SCP) components. Major functions include the survivor’s medical information, symptom journaling and tracking tool, reminders and appointments, tailored tips, and audiotaping tools.	The thematic analysis of usability testing sessions showed that the SmartSurvivor app is informative, useful in symptom tracking and communication, and portable. Interoperability with other systems, e.g., EHR, and tailoring to individuals was emphasized. All testers owned a mobile phone and regularly accessed the Internet on the mobile phone to access info, including health info.	The key finding is that the proposed app is feasible and acceptable. Key lessons learned include: the need to simplify data input and improve data output via charts and build interoperability with other systems, e.g., EHR or MyFitnessPal app.
13. Lally (2018) Oncol Nurs Forum 39	Feasibility study of researcher-developed web-module (Caring Guidance) through online focus groups (OFGs)	Rural breast cancer survivors living in rural Nebraska (n=23) recruited through flyer distributed by three cancer centers throughout Nebraska. Email and Internet access was required to join the study. iPads were available for loans as needed.	Psychological issues: Depressive-symptoms, anxiety, adjustment disorder, post-traumatic stress	CaringGuidance™ is a psychoeducational, web-based distress self-management program based on the theories of stress/coping, coping behavior, and cognitive processing and a grounded theory of acclimating to Breast Cancer. It contains six modules (22 subtopics) of supportive psycho-oncology based education/cognitive and behavioral techniques directed toward the initial months after diagnosis of early stage breast cancer.	A total of 38 women were screened and 23 (60.5%) enrolled. All women completed their assignments in OFGs. All reported having computers and Internet access at homes. The average online access to CaringGuidance is 12 days (range=1–27 days). The study identified that it is feasible to recruit and retain rural breast cancer survivors into OFGs and test a psychoeducational self-management program. The participants predominantly endorsed CaringGuidance. All participants reported having Internet access at home. Majority also reported having a mobile device.	Study identified that it is feasible to recruit and retain rural breast cancer survivors of various ages, diagnosis, and time since diagnosis for module use and OFG. In focus groups, CaringGuidance was recognized as being quality, trustworthy, relevant, easy to and comfortable to use.
14. Syrjala (2018b), J. of Cancer Survivorship^24^ Syrjala (2018a), Biology of Blood and Marrow Transplantation 23	An online, 3-arm RCT testing a web-based health information system, called and telehealth calls	Among 337 hematopoietic cell transplant (HCT) survivors with high depression, distress, and fatigue, 66 (20%) were rural participants. Email and Internet access was required to join the study.	Distress, depression, declining physical function, and fatigue.	Internet-based Survivorship Program with Information and REsources (INSPIRE): A mobile-enabled, web-based system that offers 7 levels of services, including tailored health information (e.g., self-care tips), an online forum, and secure messages to staff. Problem-Solving Treatment (PST) Telehealth Calls: Trained PhD psychologists conducted 4–8 problem solving calls sessions to help study participants identify problems, setting goals, and working forward solutions.	Among 1755 HCT survivors approached, 1306 were eligible. 205 (12%) were excluded because of having no email or computer access. The study recruitment rate is 58% (755/1306). Among 222 patients with the access to INSPIRE, the median page view is 9 (ranged 0–179). A third of them did not view pages after the homepage.^24^ About 15% of patients with the access to PST declined calls but continued to use INSPIRE. On average, PST patients receive 4.5 calls.^24^ 77% used INSPIRE at least once. Those aged 40 years and older, female, having chronic graft-versus-host disease and less than 10 years post-transplant and have moderate distress are more likely to use INSPIRE more.^23^	No statistically significant treatment effect was found in the main group comparison at 6 months after randomization.^24^ Sub-group comparison showed improved distress among INSPIRE+PST (RR=2.3, p=0.032), those viewing two or more pages in INSPIRE+PST (RR=2.7, p=0.009), and 40 and older survivors in INSPIRE+PST (RR=4.2, p=0.003) and the INSPIRE Only (RR=3.9, p=0.006).^24^ System engagement did not differ by rural vs. urban.^23^
15. McCarthy (2018) Oncology Nursing Forum 40	Prospective Pre-/post-test quasi experimental feasibility design	Participants must reside in a rural/frontier Colorado county. N=18 women with stage 1 to 3 breast cancer. Participants were required to have a computer with Internet access (could use public computers).	Insomnia and sleep outcomes	A six-week, nurse-led telemedicine-delivered Cognitive Behavioral Therapy for Insomnia (CBTI) via Adobe Connect platform (videoconferencing). The first two sessions included education on insomnia and the main components of CBTI: sleep restriction, stimulus control, and sleep hygiene, and cognitive therapy.	The enrollment rate was 82% (18/22). All 18 enrolled breast cancer survivors (BCSs) completed the study. All reported having adequate computers and Internet. No session was canceled due to technical issues. A nurse-administered, telemedicine-delivered CBTI is feasible to treat rural BCSs with insomnia. Sufficient access to high speed Internet existed and supported tele-intervention.	These survivors had significant improvement (p=0.001) in all sleep outcomes immediately after CBTI intervention, consistent with in-person CBTI findings. Participants reported a significant increase in Quality of Life (QOL), especially in emotional and function subscales (p=0.001).
16. Shinn (2019) Journal of Internet Inventions^41^	Feasibility and interim analysis of a cohort study	49.4% of Head and Neck Cancer patients (n=160) were from rural areas.	Adherence to swallowing exercises and swallowing related symptoms, such as nausea.	PREPARE is a web-based, self-management psychosocial intervention aimed at patient adherence to swallowing exercises during radiation therapy for pharyngeal and laryngeal cancers. PREPARE provides symptom reporting, swallowing exercise videos and self-management adherence and coping strategies. Participants assumed to have Internet access - were not provided with any kind of device	167 were invited and 160 (96%) were enrolled. 95 (59%) provided the 10-week adherence data. 132 (82.5%) have visited the PREPARE website. Average number of site visits was 5.49 (SD=9,95) over the 10-week intervention period. The average website visit was 5.5 (SD=9.96) and time spent was 41 mins (SD=88.5) over 10 weeks. A general declining trend on usage was found.	Of those reporting adherence, 51–53% were adherent to preventive exercises. The number of unique visits to the PREPARE website is related to increased adherence to preventive exercise (p=0.001–0.008).
17. Gilberston-White (2019) The Journal of Rural Health 42	3-phase mixed methods used to (1) assess stakeholder needs and opinions, (2) develop a symptom self-management website, and (3) obtain usability feedback from potential users.	Rural solid tumor patients (n=16) and clinicians (n=10) for ethnographic review, and rural patients (n=126) and family members (n=52) for usability testing. Patients were recruited from rural medical oncology clinic, rural radiation oncology clinic, and tertiary care medical center with large rural referral.	Anxiety, constipation, depression, diarrhea, drowsiness, fatigue, lack of appetite, memory problem, mouth sores, pain, nausea, tingling, shortness of breath, skin/nail changes, and sleep disturbance	Oncology Associated Symptoms and Individualized Strategies (OASIS) web application consisting of 1) patient educational material about common cancer symptoms, 2) a daily tracker for user to record their symptom distress and use of symptom management strategies, and 3) a personal dashboard that charts symptom distress scores and daily strategies https://oasis.nursing.uiowa.edu/aboutoasis A weekly nurse-coach video conferencing call was added to provide tailored management strategies and currently tested in another study.	63.5% of participants reported using the Internet multiple times per day. Only 4% have never used the Internet. Participants with limited access to or not using Internet/technology have other walkaround solutions, such as via borrowing from family members or ask family members to use on their behalf. Usability testing with 126 stakeholders demonstrated that the web application was easy to use, contains relevant content, and has pleasing graphics. Time spent on OASIS was 10 to 90 minutes. No differences found in learnings among different user types (patients, family/caregiver, staff)	No impact on the outcomes of patients, families, communities, or health systems was reported. Technology use in this population was nearly universal. Rural stakeholders reported significant challenges with cancer symptom management and were extremely interested in using technology to address those challenges. While some liked the idea of self-management, others found their symptoms overwhelming and the process of self-management was daunting.

Note: Abbreviations: ES: Effect Size, RR: Relative Risk.
